# Ion adsorption-induced wetting transition in oil-water-mineral systems

**DOI:** 10.1038/srep10519

**Published:** 2015-05-27

**Authors:** Frieder Mugele, Bijoyendra Bera, Andrea Cavalli, Igor Siretanu, Armando Maestro, Michel Duits, Martien Cohen-Stuart, Dirk van den Ende, Isabella Stocker, Ian Collins

**Affiliations:** 1 University of Twente, MESA+ Institute for Nanotechnology, Physics of Complex Fluids, P.O. Box 217, 7500AE Enschede (The Netherlands); 2 BP Exploration Operation Company Ltd., Chertsey Road, Sunbury-on-Thames, TW16 7LN, (United Kingdom)

## Abstract

The relative wettability of oil and water on solid surfaces is generally governed by a complex competition of molecular interaction forces acting in such three-phase systems. Herein, we experimentally demonstrate how the adsorption of in nature abundant divalent Ca^2+^ cations to solid-liquid interfaces induces a macroscopic wetting transition from finite contact angles (≈10°) with to near-zero contact angles without divalent cations. We developed a quantitative model based on DLVO theory to demonstrate that this transition, which is observed on model clay surfaces, mica, but not on silica surfaces nor for monovalent K^+^ and Na^+^ cations is driven by charge reversal of the solid-liquid interface. Small amounts of a polar hydrocarbon, stearic acid, added to the ambient decane synergistically enhance the effect and lead to water contact angles up to 70° in the presence of Ca^2+^. Our results imply that it is the removal of divalent cations that makes reservoir rocks more hydrophilic, suggesting a generalizable strategy to control wettability and an explanation for the success of so-called low salinity water flooding, a recent enhanced oil recovery technology.

The relative wettability of oil and water on porous solids is crucial to many environmental and technological processes including imbibition, soil contamination/remediation, oil-water separation, and the recovery of crude oil from geological reservoirs^1^,^2^,^3^,^4^,^5^,^6^,^7^. Good wettability of a porous matrix to one liquid generally implies stronger retention of that fluid and simultaneously easier displacement of the other. In standard ‘water flooding’ oil recovery, (sea) water is injected into the ground to displace oil from the porous rock, typically at an efficiency <50%. For decades, oil companies have explored adding chemicals such as surfactants and polymers to the injection water to improve the process^8^,^9^. More recently, it was discovered that the efficiency can also be improved by reducing the salinity of the injection water^10^, *i.e.* without adding expensive and potentially harmful chemicals, known as low salinity water flooding (LSWF). Yet, reported increases in recovery vary substantially and the microscopic mechanisms responsible for the recovery increment remain debated^9^,^11^,^12^,^13^. A wide variety of mechanisms has been proposed to explain the effect, including the mobilization of fines, interfacial tension variations, multicomponent ion exchange, and double layer expansion^10^,^11^,^12^,^14^. Many of these mechanisms are interrelated and may ultimately result in improved water wettability of the rock but evidence discriminating between them is scarce. The key challenge in identifying the reasons for the success of LSWF lies in the intrinsic complexity of the system and the lack of direct access to its microscopic properties. Here, we experimentally demonstrate for a well-defined model system a consistent scenario leading from ion adsorption at the solid-liquid interface to charge reversal and from there to wettability alteration. We also derive a model that provides quantitative predictions of the experimentally observed contact angles. Our results clarify many previous observations in core flooding experiments, including in particular the relevance of divalent cations, clays, pH, and polar organic species.

## Results & Discussions

### Wettability alteration

**Figure 1:**
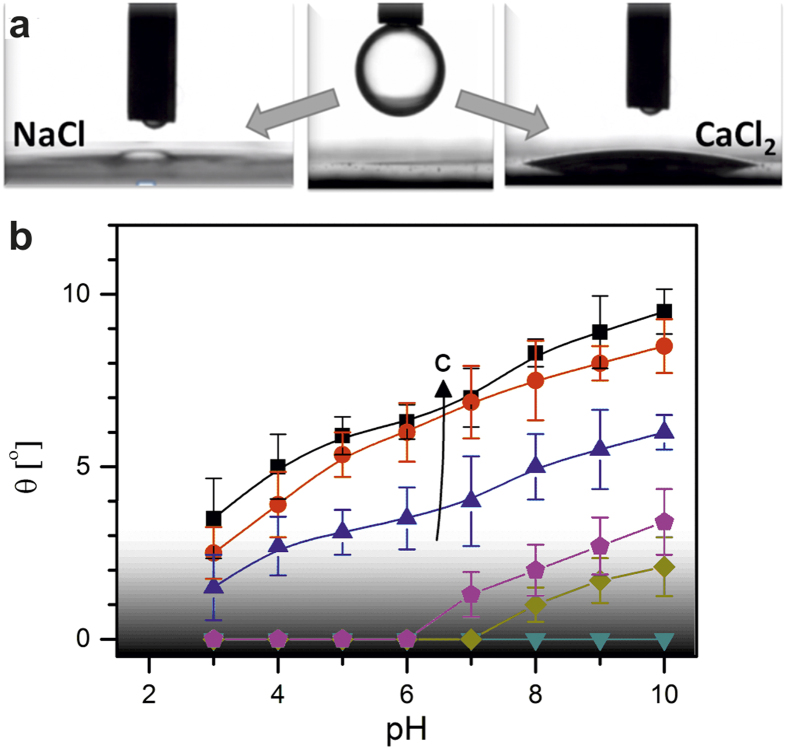
Water wetting on mica in ambient decane for monovalent & divalent salt solutions. ** (a)** Side view of drops of 1 M (pH 7) aqueous solutions of NaCl (left) and CaCl_2_ (right) immediately after bringing the drop on the needle in contact with the mica surface (ambient fluid: decane; needle diameter: 0.5 mm). NaCl solutions display immeasurably small contact angles, CaCl_2_ solutions can display a finite contact angle, depending on concentration and pH. (**b)** Symbols: Equilibrium contact angle on mica vs pH for CaCl_2_ salt solutions of various concentrations; 1,10,30 mM (downward triangles), 50 mM (olive diamonds), 80 mM (purple pentagons), 100 mM (blue triangles), 500 mM (red circles), 1 M (black squares). Solid lines: guides to the eye. The shaded region indicates very low contact angles, which are close or below the sensitivity of the instrument. The arrow with the letter c denotes the direction of increasing salt concentration.

The rock of common sandstone reservoirs consists of highly polar materials such as quartz and clays that in ambient air are completely wetted by both water and oil. To analyze the competitive wetting of oil and water on these substrates, we measured the macroscopic contact angle of water of variable salt content against decane. We chose flat, freshly cleaved mica and freshly cleaned silica surfaces as model materials, to represent the basic components of sandstone reservoirs. The macroscopic contact angle of water as observed in side view images, Fig. 1a, on mica and silica in ambient decane was found to depend strongly on the composition of the aqueous phase. We varied pH between 3 and 10 and concentrations of NaCl, KCl and CaCl_2_ from 1 mM to 1 M (see Methods). Aqueous drops containing monovalent cations invariably spread to immeasurably small contact angles (<2°); in contrast, drops containing divalent cations displayed finite contact angles on mica for concentrations above ≈50 mM and pH > 4 (Fig. 1; see also Supplementary Material, movies S1 and S2). On silica, negligible contact angles were found for all pH’s and concentrations of all salts investigated, *i.e.* including the ones with divalent cations.

### Proposed adsorption mechanism

**Figure 2:**
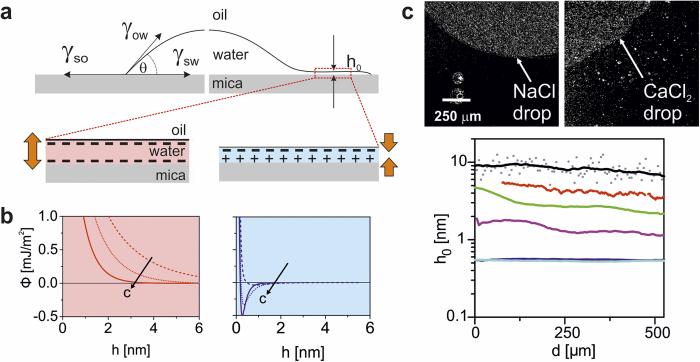
Proposed mechanism of wetting transition through ion adsorption and charge reversal at mica-water interface. ** (a)** Schematic view of force balance, thin film with equilibrium thickness h_0_ (top) and surface charge configurations of repulsive (bottom left) and attractive interface potential (bottom right). (**b)** Effective interface potential for surface charges of equal (left, red lines, mica-NaCl solution at pH 6-oil) and of opposite sign (right, blue lines, mica-CaCl_2_ solution at pH 6-oil), leading to near-zero and finite contact angles, respectively. Lines denote salt concentrations: 1 mM (dashed lines), 10 mM (dotted lines) & 100 mM (solid lines). The arrows with the letter c denote the direction of increasing salt concentration. (**c)** Ellipsometry images (top) and resulting thickness profiles (bottom): film thickness (h_0_) vs distance from contact line for aqueous drops for various concentrations of CaCl_2_: 1 M (dark blue), 500 mM (magenta), 100 mM (green) and 10 mM (red) at pH 6; 1 M NaCl (black; grey symbols indicate scatter of raw data). Light blue: water film thickness in decane before adding aqueous drop.

To identify the origin of the wetting transition on mica, we analyzed the force balance between the decane-water (γ), solid-decane (γ_so_) and solid-water (γ_sw_) interfacial tension at the three phase contact line. Under partial wetting conditions, the spreading pressure 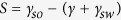
is negative and the equilibrium contact angle θ (measured through the aqueous phase) is given by Young’s equation (Fig. 2a), 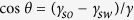
15. For water in contact with non-polar oils, γ depends very weakly on pH and salt content (Supplementary Figure 1) and hence has a negligible influence on the wettability^16^. γ_sw_ usually decreases as salt content increases due to the spontaneous formation of an electric double layer at the solid-water interface^17^. Because any *reduction* of γ_sw_ can only induce a decrease of θ , the observed increase upon addition of Ca^2+^ and Mg^2+^ ions must be caused by an even stronger decrease of γ_so_. The latter is plausible if the system forms a nanometer thin aqueous film next to the macroscopic drop with a salinity-dependent thickness h_0_ (Fig. 2a). Using imaging ellipsometry we indeed detected such a film, as shown in Fig. 2c. Upon increasing the CaCl_2_ concentration, h_0_ decreased from approximately 8 nm to less than 1 nm. For pure water and for NaCl solutions, ellipsometry measurements revealed that θ is very small but finite despite the apparent spreading in side view images; h_0_ was found to be ≈ 10 nm. Given the existence of this nanofilm, we can write the equilibrium tension γ_so_ in terms of oil-water and solid-water interfacial tensions plus an effective interface potential Φ(h) representing the molecular interactions between the solid-water and the water-oil interface as^15^ 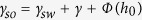. Here, Φ(h_0_) is the equilibrium value of Φ(h) corresponding to the equilibrium film thickness h *= *h_0_, such that


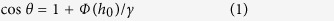


The ion-induced wettability alteration thus reflects the salt-dependence of Φ(h), Fig. 2b.

### Interfacial charge reversal

We decomposed 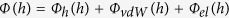 into contributions from short-range chemical hydration forces 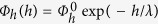, van der Waals forces 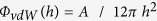, and electrostatic forces Φ_el_(h). While the amplitude 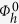 and the decay length λ of the repulsive hydration forces as well as the Hamaker constant A generally vary weakly with pH and salt concentration, they are not expected to change sign for the conditions of our experiments^18^. Hence, we conclude that the observed wettability alteration is driven by Φ_el_(h). The latter is repulsive and thus favors complete wetting if the charge densities σ_sw_ and σ_ow_ of the solid-water and the oil-water interface, respectively, carry the same sign. Vice versa, surface charges of opposite signs result in attraction and partial wetting. σ_sw_ and σ_ow_ are thus key parameters controlling wettability, as recently recognized in the context of wetting transitions with electrolyte solutions^19^,^20^.

**Figure 3:**
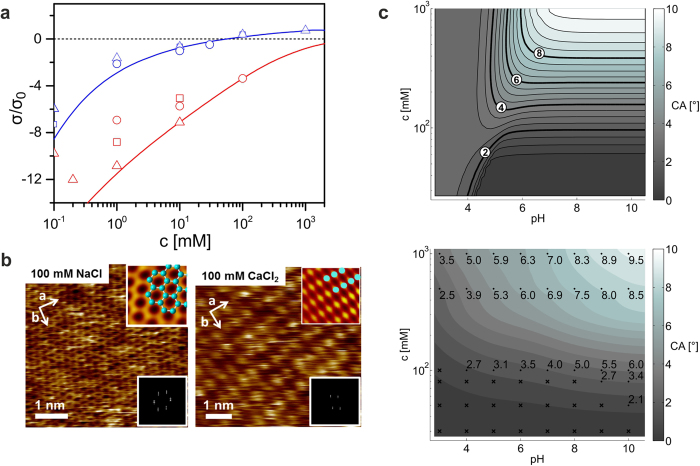
Ion adsorption at mica-water interface. (**a)** Surface Charge calculated from ζ potential measurements (circles) vs. concentration of solutions of NaCl (red) and CaCl_2_ (blue) at pH 6. Solid lines: surface complexation model predictions. Blue triangles: AFM data from^25^; blue squares^24^, red triangles^23^, red squares^32^: surface forces apparatus measurements. The charge density is normalized by the characteristic scale 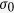 arising from the Poisson-Boltzmann equation, 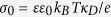, where 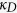 is the Debye parameter. (**b)** AFM images of mica-water interface showing the characteristic hexagonal lattice of mica in 100 mM NaCl solution (left), and a rectangular symmetry caused by (presumably hydrated) adsorbed Ca^2+^ ions in 100 mM CaCl_2_ (right). Insets: filtered zoomed views with overlaid lattice structure (top) and Fast Fourier Transform image of the same data (bottom). **c** Gray scale encoded contact angle vs. pH and CaCl_2_ concentration. Top: model prediction; bottom: experimental data. Symbols (x: θ < 2°) and numbers: experimental data same as Fig. 1b with interpolated gray scale. Smoothed lines are guides to the eye based on the experimental datapoints.

For oil-water interfaces, σ_ow_ is negative for pH > 3. The adsorption of ions is rather weak^21^,^22^, as we corroborated using streaming potential measurements with solid eicosane mimicking decane. In streaming potential measurements for NaCl and KCl solutions, negative surface charges prevailed on mica for all conditions investigated, in agreement with surface force measurements^17^,^23^. For CaCl_2_, however, a much stronger adsorption was found, Fig. 3a, leading to charge reversal at concentrations beyond ~ 50 mM^24^. Atomic force microscopy (AFM) confirmed this distinct difference between monovalent and divalent cations. While AFM images in pure water and aqueous NaCl and KCl solutions displayed the intrinsic hexagonal appearance of bare mica, a transition to a rectangular pattern was found for ambient CaCl_2_ solutions, Fig. 3b^25^. Similar to gibbsite-water interfaces^26^, we attribute this pattern to a layer of strongly adsorbed, possibly hydrated, divalent cations that reverse the sign of σ_sw_.

### Interaction between interfaces

To quantitatively assess this suggested mechanism, we explicitly calculate the various contributions to the disjoining pressure discussed in the previous section. Φ_h_(h) is characterized by an amplitude 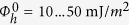 and a decay length 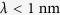
25,27. For Φ_vdW_(h) we use a Hamaker constant 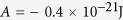 limited by the experimental constraint that the finite contact angle of NaCl and KCl solutions must not exceed 2°. This negative Hamaker constant implies long range partial wetting, which arises from the fact that water has a lower refractive index than both mica and oil. We obtain the electrostatic contribution Φ_el_ to the disjoining pressure by solving the Poisson-Boltzmann equation for the electrostatic potential 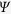 inside the thin film, which reads 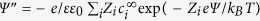. In the equation, 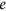 is the elementary charge, 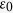 and 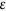 are the vacuum and relative permittivity of the medium and 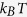
is the thermal energy in the system. The sum runs over the ions in the solution, with 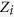 representing the valence and 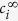 the bulk concentration of the i-th specie. Here, we have used the full Poisson Boltzmann expression instead of classical examples^28^,^29^ of a reduced equation, since the zeta potentials in our system are clearly beyond 25 mV. We apply constant charge (CC) boundary conditions, where the surface charges σ_sw_ (at the solid-water interface) and σ_ow_ (at the oil-water interface) are determined from the corresponding surface complexation model (see Methods), by fitting to experimentally measured streaming potentials. Once the electrostatic potential 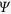 is known, we find the contribution to the disjoining pressure Φ_el_ by evaluating the standard expression 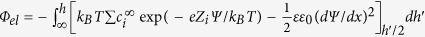 ^18^.

Adding up all the contributions to the disjoining pressure, we find that for sufficiently high Ca^2+^ concentrations, Φ(h) indeed develops a pronounced minimum at small h_0_, corresponding to water contact angles up to 10°, as depicted in Fig. 2b. For Na^+^ and K^+^, however, a very shallow minimum corresponding to a small but finite contact angle appears, due of the dominance of attractive van der Waals interactions (*i.e.* 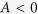) for large film values of h.

Using eq. (1), we extracted the contact angle θ from the minima of Φ(h) for all fluid compositions, Fig. 3c,top. Comparison to the experimental results, Fig. 3c,bottom, shows that the model indeed captures all salient features of the experiments, including in particular the transition from near zero contact angles at low divalent ion concentration and pH to values of 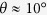 at high Ca concentration and pH. For monovalent cations on mica and for all salts on silica, the same calculation invariably results in repulsive electrostatic forces and hence negligibly small contact angles (<2°).

### Synergistically enhanced wettability alteration

**Figure 4:**
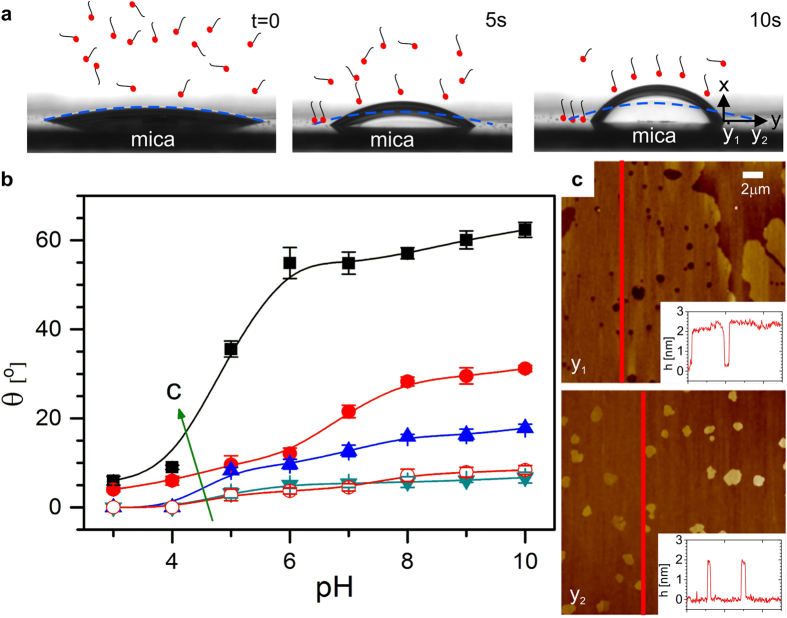
Cation-induced surfactant adsorption on solid substrate in oil. ** (a)** Snapshots of drops of 1 M CaCl_2_ solution (pH = 9) on mica immersed in ambient decane containing 100 μM stearic acid, immediately after deposition (t = 0) and 5 s and 10 s later. Drops display autophobic behavior due to the deposition of organic layers on the substrate. (**b)** Equilibrium contact angle vs. pH for various concentrations of CaCl_2_: 1 mM (cyan downward triangles), 10 mM (blue upward triangles), 100 mM (red circles), 1 M (black squares) and NaCl: 100 mM (red open circles). The arrow with the letter c denotes the direction of increasing salt concentration. Stearic acid concentration: 100 μM. (**c)** After drop removal and drying AFM images display an almost complete monolayer at a distance of y_1_ = 100 μm from the original contact line and an almost bare substrate with occasional stearate islands at y_2_ = 800 μm. Height profiles, corresponding to the red lines in the AFM images, demonstrate that the thickness of the layer corresponds to the length of a stearate monolayer.

Most crude oils contain small proportions of surface-active polar components in addition to alkanes. We investigated the impact of these components on the wettability by adding small amounts of stearic acid (S.A.) to the decane. Water drops containing divalent cations, when deposited on mica under decane/S.A. mixture, initially assumed 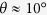, as in absence of S.A. Within seconds, however, θ increased to values of up to 70° (Fig. 4a,b; Movie S3). For drops containing NaCl, θ slightly increased, too, but never exceeded 10°. AFM imaging of the mica surface after removal from all liquids revealed the origin of this strong autophobic behavior: the surface was covered by a stearate monolayer very similar to partially decomposed Langmuir-Blodgett films of the same material reported earlier^30^. Close to the original contact line of the droplet, this layer was dense with occasional holes; farther away, bare mica was seen with occasional islands of monolayer stearate. Ca^2+^ and S.A. thus synergistically enhance the wettability alteration by promoting the self-assembly of hydrophobic Ca stearate monolayers.

In conclusion, these findings demonstrate how divalent cations in combination with clays and acidic components in the oil can control the wettability of oil-water-rock systems in water flooding oil recovery. The observed reduction in the water-mica contact angle in ambient decane of approximately 10°, as a result of removing divalent ions from the water, is itself sufficient to result in several percent of incremental oil recovery^31^. More generally, our results suggest a universal strategy to manipulate wettability by controlling the adsorption of ions to solid-liquid interfaces.

## Methods

### Experimental System

Anhydrous n-decane (>99%, Sigma Aldrich) is passed five times through a vertical column of Alumina powder (Al_2_O_3_, Sigma Aldrich, Puriss grade > 98%) to remove any surface-active impurities. The ultrapure water (resistivity 18 MΩ) used to prepare the salt solutions is obtained from a Millipore water treatment system (Synergy UV Instruments). Solutions of various concentration (between 1 mM to 1 M cation concentration) are prepared for NaCl, KCl or CaCl_2_ salts (Sigma Aldrich). The pH of the solution is adjusted between 3 and 10 using HCl/HNO_3_ and NaOH (0.1 M, Sigma Aldrich). Muscovite mica (B&M Mica Company Inc., USA; initial thickness 340 μm) and oxidized silicon wafers with an amorphous silicon oxide layer (thickness: 30 nm) mimicking silica represent the surface of a solid rock. Mica sheets are cleaved inside the oil phase to obtain a pristine surface during the experiment. Silica surfaces are cleaned using a combination of Piranha solution (followed by extensive rinsing with ultrapure water) and plasma treatment.

### Contact angle measurements

The wetting of aqueous drops on mica is characterized using a commercial contact angle goniometer (OCA 20L, Dataphysics Inc.). The measurement is based on sessile-drop method using aqueous drops with a volume of 2 μL placed on solid substrate. The contact angle of the drops is extracted from video snapshots using the tangent-fitting method in data analysis software (SCA 22) provided with the instrument. Contact angles can be determined with a relative accuracy of ±1°. The minimum contact angle that can be determined on reflective surfaces is approximately 1.5°. Before placing the aqueous drops on the substrates, pendant drop measurements are performed to determine the oil/water interfacial tension (IFT). Constancy of the IFT over time ensures that the oil is devoid of residual surface active contaminants after passing the alumina powder column.

### Ellipsometry

Thickness measurements of ultrathin wetting films were performed using an imaging ellipsometer (Accurion). The ellipsometer is equipped with custom-built quartz tubes attached to both the source (laser) and the detector arm to enable measurements under liquid at variable angle of incidence. In the case of mica, the bottom side of the substrate was roughened and coated with an index matched epoxy resin to suppress interference. Null ellipsometry experiments were performed. The thickness *h*_0_ of the potentially adsorbed water layer is extracted from the ellipsometric angles Ψ and Δ assuming the bulk refractive index of the adjacent aqueous drop using standard Fresnel coefficients for a three layer system (substrate –water–oil).

### Zeta Potential measurement

Surface charge and surface potential of solid/water (or oil/water) interfaces were determined by streaming potential measurements using a ZetaCAD instrument (CAD Instruments, France). The measurement cell consists of two substrates of the solid under investigation (50 mm x 30 mm) at a separation of 100 μm. Measured ζ potentials are converted to (diffuse layer) surface charges using Grahame’s equation.

### Surface complexation modeling

The surface charge of solid-water interfaces is modelled using standard surface complexation models involving adsorption/desorption reactions of cations X_i_ (i = H^+^, Na^+^, Ca^2+^) to surface sites S following the scheme 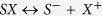. Each reaction is characterized by an equilibrium constant K with a corresponding value 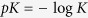. The law of mass action relates the cation concentration [X_i_]_s_ at the surface and the surface concentrations {SX} and {S^-^} to the equilibrium constant: 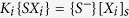. Local concentrations at the surface are related to the corresponding bulk concentrations 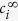 by a Boltzmann factor 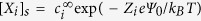, where Ψ_0_ is the potential at the surface and *Z*_i_ the valency of species *i*. For the oil-water interface, the primary charge generation mechanism is assumed to be the autolysis of water 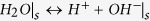
21 . Additional weak cation adsorption reactions are included, too. The surface charge is then given by the relation 
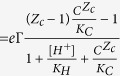
, where 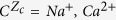 represent the activity of the ions considered. At large separation, the implicit dependence on 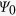 is solved equating this value to the one predicted by the Grahame Equation for monovalent 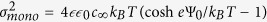 and divalent 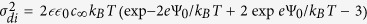 salts, respectively. We use this procedure to extrapolate the value of the surface charges for all pH and salt concentrations considered. Our choice of the equilibrium constants is based on values from literature: a complete overview of all surface reactions and pK values is provided in the supplementary information, Table S1. In Fig. 3a we observe a good agreement between the values obtained by this approach (full lines) and several experimental measurements of the surface charge of Mica for monovalent and divalent salts.

## Additional Information

**How to cite this article**: Mugele, F. *et al*. Ion adsorption-induced wetting transition in oil-water-mineral systems. *Sci. Rep.* **5**, 10519; doi: 10.1038/srep10519 (2015).

## Supplementary Material

Supplementary Information

Supplementary Movie 1

Supplementary Movie 2

Supplementary Movie 3
