# Supplementary material for: Ion adsorption-induced wetting transition in oil-water-mineral systems
Source: Sci Rep. 2015 May 27;5:10519. doi: 10.1038/srep10519 (PMC4444960; doi:10.1038/srep10519)
Supplement: Supplementary Information [file srep10519-s1.pdf]

Supplementary Information

Ion adsorption-induced wettability alteration in oil-water-mineral systems

Frieder Mugele[1], Bijoyendra Bera[1], Andrea Cavalli[1], Igor Siretanu[1], Armando Maestro[1], Michel Duits[1], Martien Cohen-Stuart[1], Dirk van den Ende[1], Isabella Stocker[2], and Ian Collins[2]

[1] University of Twente, MESA+ Institute for Nanotechnology, Physics of Complex Fluids, P.O. Box 217, 7500AE Enschede (The Netherlands).

[2] BP Exploration Operation Company Ltd. , Chertsey Road, Sunbury-on-Thames, TW16 7LN, (United Kingdom).

S1. Contribution of Interfacial tension at Oil/Water Interfaces

Oil/water interfacial tension is measured using Pendant Drop method in OCA 20 (Dataphysics Inc.). A pendant drop (volume 15-20 µl) of salt solution is prepared in ambient decane phase, and using tangent fitting to the contour of the drop, interfacial tension is measured. We observe no significant change in interfacial tension with change of pH, but with increasing concentration of salt in the aqueous phase, a small decrease in the interfacial is noticed (Supplementary Figure 1). If the interfacial tensions at solid/water and solid/oil interfaces remain constant, then Supplementary Figure 1 would suggest a decrease in contact angle with increasing salt concentration. But we notice an increase in contact angle with increasing salt concentration, which confirms our hypothesis of existence of a thin wetting film between mica and decane.

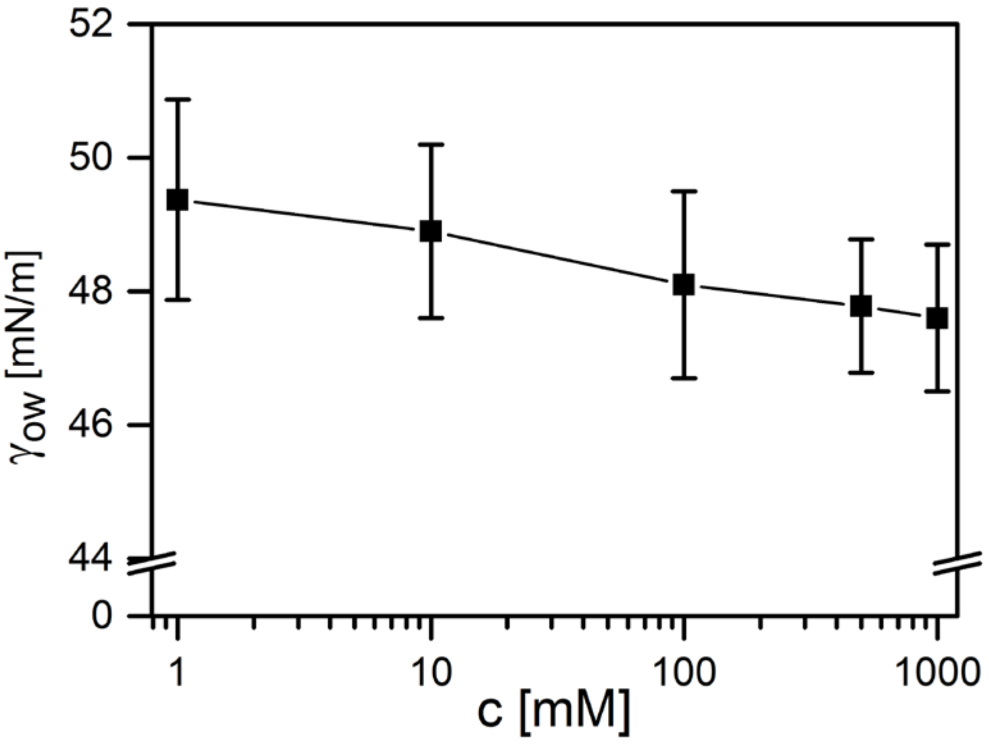

Supplementary Figure 1 | Equilibrium interfacial tensions for the decane-water interface. Squares: Interfacial tension vs salt concentration measured with a pendant drop of $CaCl_2$ salt at pH 7 in ambient decane (oil), the error bars show the standard error. Solid line: guide to the eye.

	Mica interface	Oil interface
**Reactions**	$\{SH\} \leftrightarrow \{S^-\} + [H^+]$ $\{SC^{Z_c}\} \leftrightarrow \{S^-\} + \langle C^{Z_c}\rangle$ $\{SC^{Z_c}\} + \{S^-\} + \{SH\} = \Gamma_{\text{mica}}$	$\{SH_2O\} \leftrightarrow \{SOH^-\} + [H^+]$ $\{SOH - C^{Z_c}\} \leftrightarrow \{SOH^-\} + \langle C^{Z_c}\rangle$ $\{SH_2O\} + \{SOH^-\} + \{SOH - C^{Z_c}\} = \Gamma_{\text{oil}}$
**Coefficients**	$\Gamma_{\text{mica}} = 0.8 \text{ sites/nm}^2$ $PK_{Ca^{2+}} = 1.5$ $PK_{Na^+} = 0.5$ $PK_{H^+} = 5.3$	$\Gamma_{\text{oil}} = 17.3 \text{ sites/nm}^2$ $PK_{Ca^{2+}} = -1$ $PK_{Na^+} = -1$ $PK_{H^+} = 7$
**Charge**	$\sigma = e\Gamma \frac{(Z_c - 1)\frac{\langle C^{Z_c}\rangle}{K_C} - 1}{1 + \frac{[H^+]}{K_H} + \frac{\langle C^{Z_c}\rangle}{K_C}}$	$\sigma = e\Gamma \frac{(Z_c - 1)\frac{\langle C^{Z_c}\rangle}{K_C} - 1}{1 + \frac{[H^+]}{K_H} + \frac{\langle C^{Z_c}\rangle}{K_C}}$

Supplementary Table 1 | Complexation model coefficients for the reactions at the Mica-water interface (left) and Oil-water interface (right). { } represents surface concentrations, [ ] bulk concentrations and < > the bulk activity of a given specie.

[Movie S1]

Movie S1 | 'Almost' complete wetting with NaCl. Typical behaviour of an aqueous drop (volume 2µl) containing a monovalent salt (NaCl) at any concentration or pH. The drop spreads on the mica (which is in ambient decane) without forming any wedge-shaped drop, thus making impossible to characterize a contact angle with the optical goniometer.

[Movie S2]

Movie S2 | Partial wetting with $CaCl_2$. An aqueous drop (volume 2µl) containing divalent $CaCl_2$/$MgCl_2$ solution (at a concentration higher than 50mM) does not spread on mica (in ambient decane) as in the previous case. A wedge-shaped drop is formed, where a small but definite finite contact angle is possible to measure with the goniometer.

[Movie S3]

Movie S3 | Synergistic contact angle enhancement, with $CaCl_2$ and added stearic acid to the oil phase. An aqueous drop (volume 2µl) containing divalent $CaCl_2$ solution (at a concentration of 1M) is deposited on mica in ambient decane. 0.1mM stearic acid is added to the oil phase. The water droplet forms a finite but small contact angle, which grows rapidly due to the autophobing effect described in the main manuscript.
